# Supplementary figures and images for: SUMO-mediated regulation of H3K4me3 reader SET-26 controls germline development in C. elegans
Source: PLoS Biol. 2025 Jan 6;23(1):e3002980. doi: 10.1371/journal.pbio.3002980 (PMC11703099; doi:10.1371/journal.pbio.3002980)

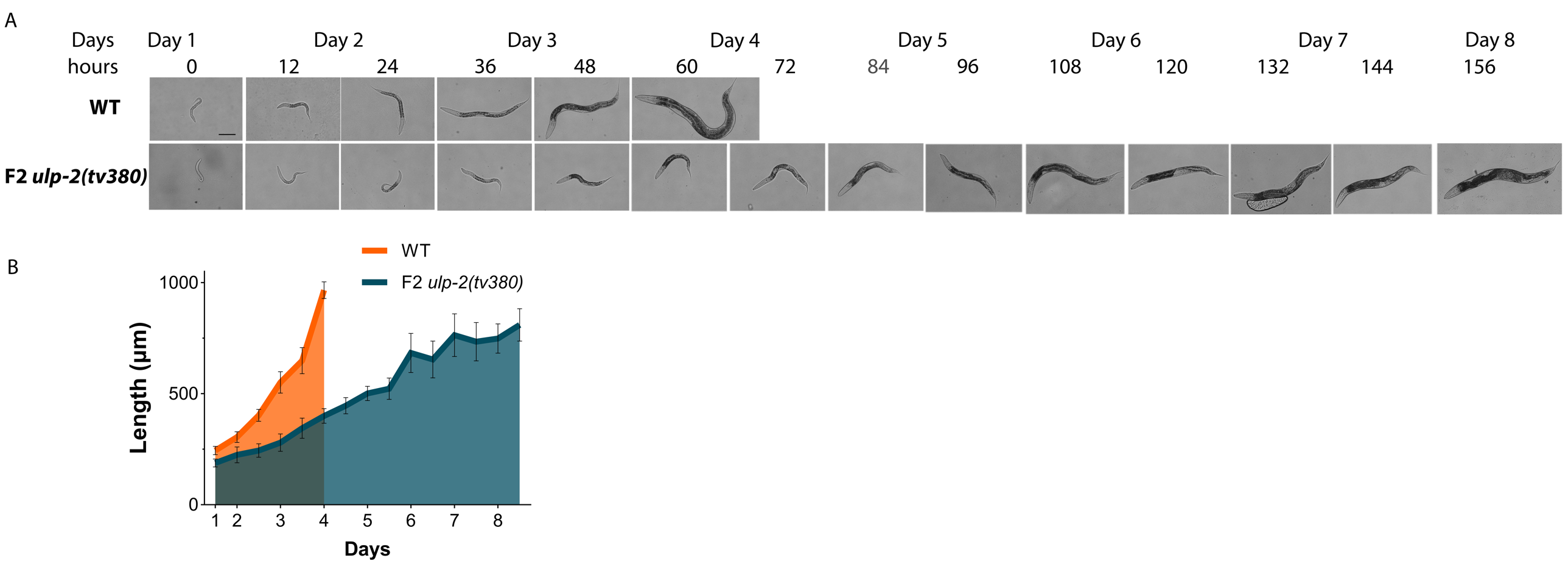

Supplement: S1 Fig — (A) Representative images of WT and ulp-2(tv380). Imaging started upon hatching. Animals were grown in 20°C and imaged every 12 h. Scale bar = 100 μm. (B) Mean length of animals (μm) at each time point (n = 10 for each time point and genotype). The numerical data presented in panel B can be found in S1 Data. (TIF) [file pbio.3002980.s001.tif]

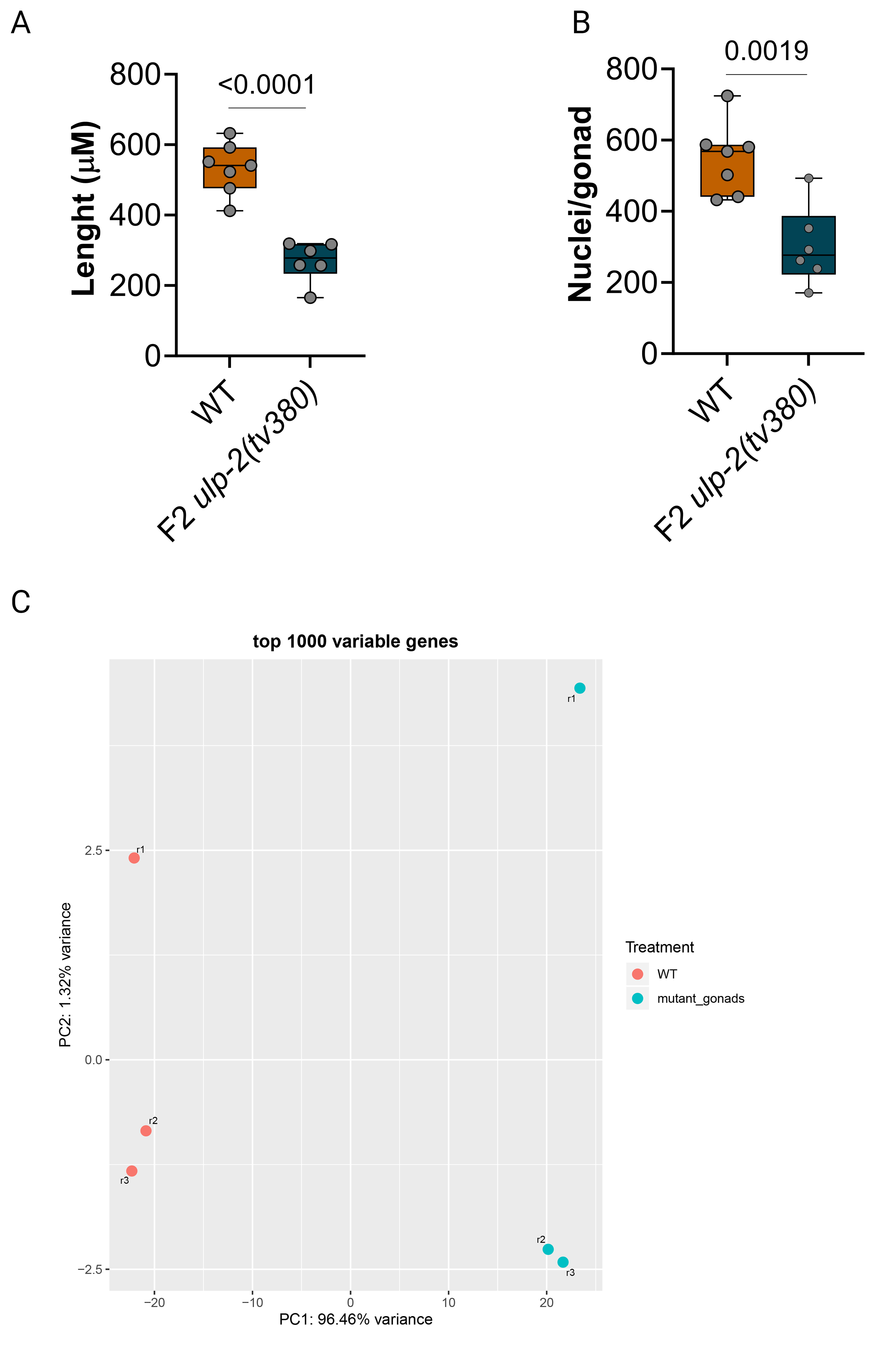

Supplement: S2 Fig — (A) Quantification of germline length of WT (7 germlines) and F2 ulp-2(tv380) (6 germlines); two-tailed Welch’s t test was used, ns = p > 0.05. (B) Quantification of the total number of nuclei in WT (3834 nuclei) and F2 ulp-2(tv380) (1809 nuclei). Two-tailed Welch’s t test was used, ns = p > 0.05. (C) PCA analysis of the RNA-seq data set of WT (3 biological samples, 779 isolated germlines) and F2 ulp-2(tv380) (3 biological samples, 990 isolated germlines); pink dots represent the biological replicates of WT isolated germlines and blue dots represent the biological replicates of F2 ulp-2(tv380) isolated germlines. The numerical data presented in this figure can be found in S1 Data and in S6 Table. (TIF) [file pbio.3002980.s002.tif]

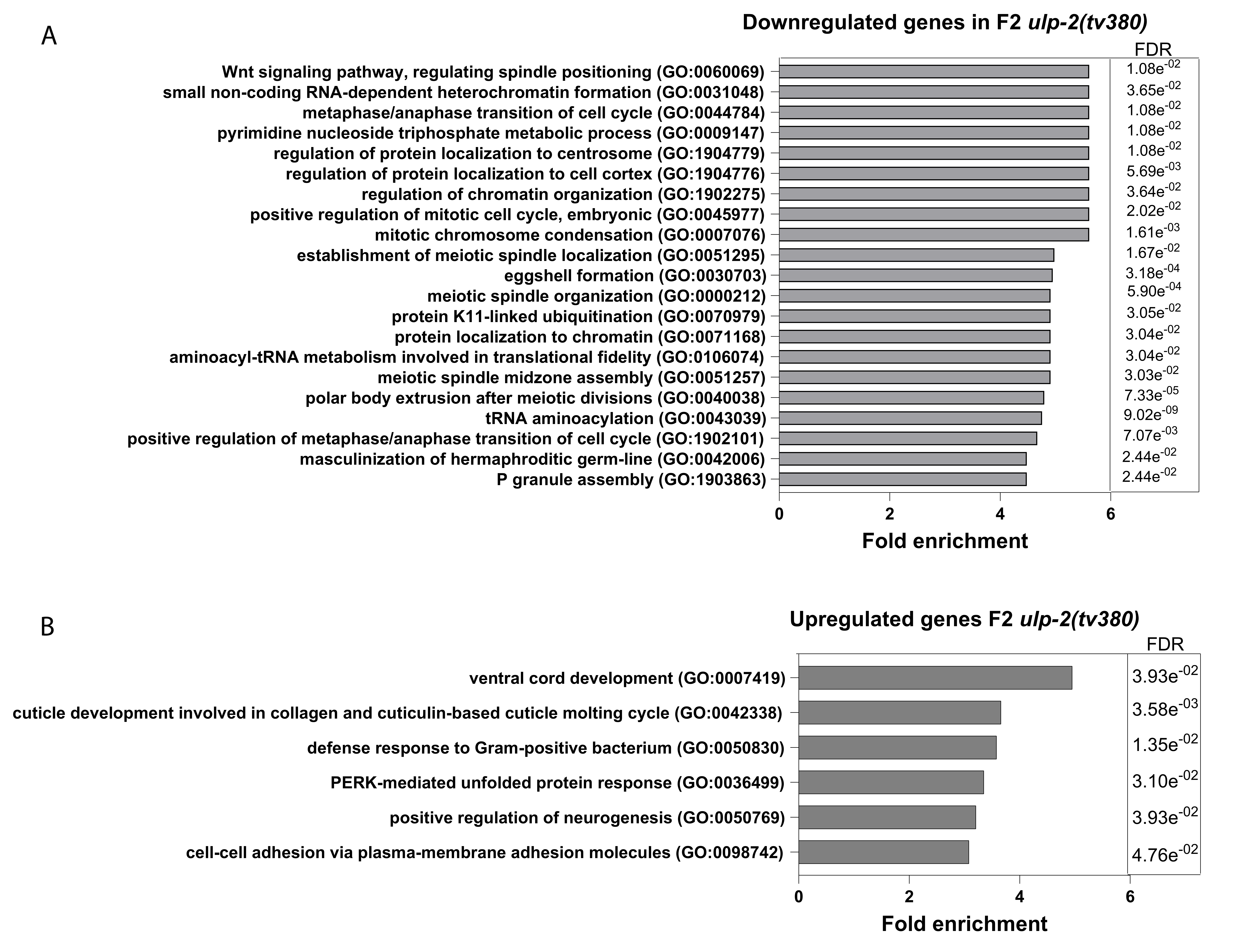

Supplement: S3 Fig — (A) Gene ontology analysis of the biological processes containing the down-regulated genes in germlines of F2 ulp-2(tv380) when compared to WT. Biological processes were cut off at Fold enrichment<4; ns = FDR > 0.05. (B) Gene ontology analysis of the biological processes containing the significantly up-regulated genes in F2 ulp-2(tv380) when compared to WT. Biological processes were cut off at Fold enrichment<2.5; ns = FDR > 0.05. The numerical data presented in this figure can be found in S2 Table. (TIF) [file pbio.3002980.s003.tif]

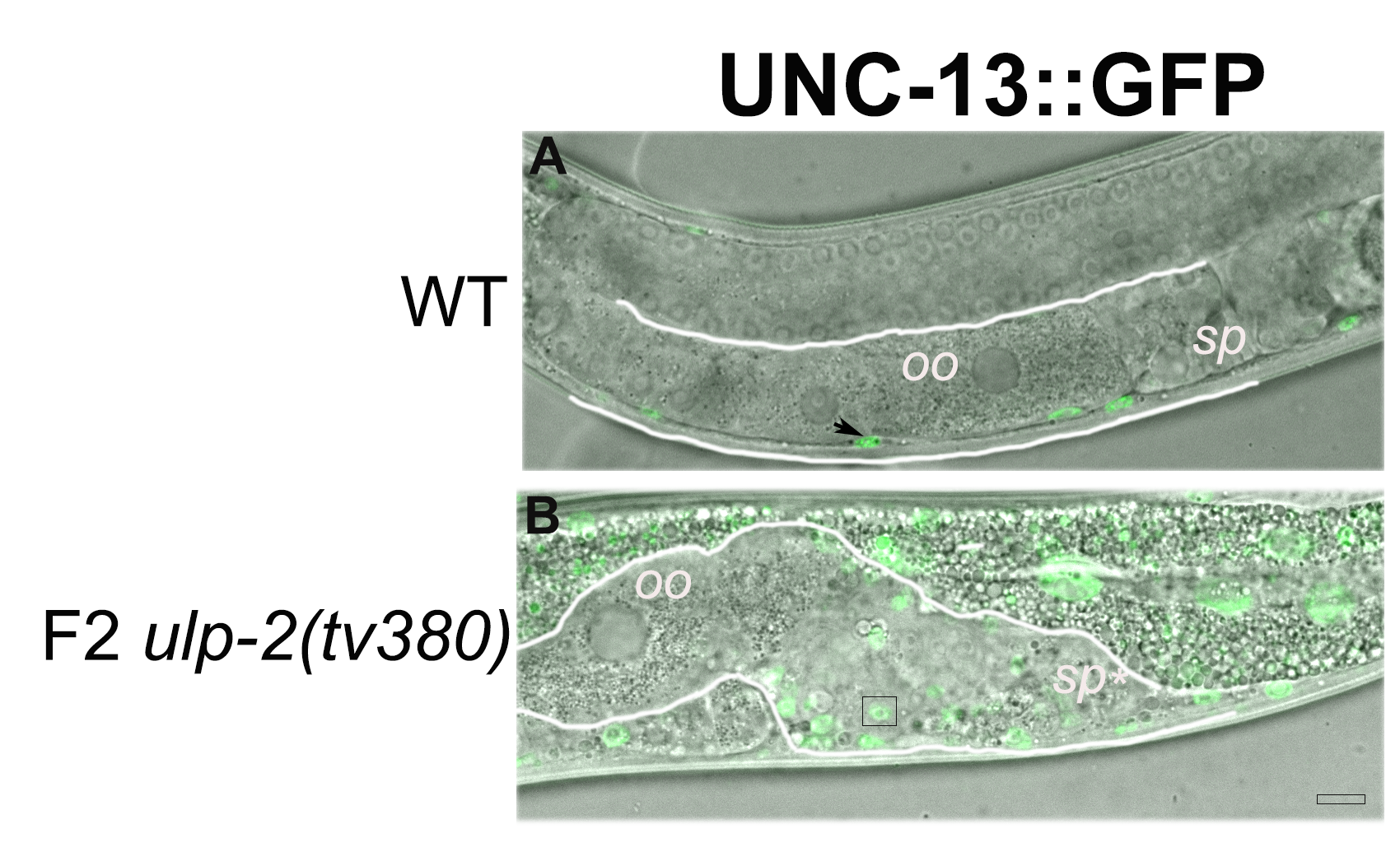

Supplement: S4 Fig — (A) In WT gonads the endogenously tagged UNC-13::GFP reporter is expressed only in the somatic gonad sheath cells (arrow) (n = 30). (B) Representative image of UNC-13::GFP localization in the germline of F2 ulp-2(tv380) animals. A cell expressing UNC-13::GFP in the germline is labeled with a black box. White line marks the proximal gonad, the most proximal oocyte (oo) and spermatheca (sp) or predicted spermatheca (sp*). (n = 32). Scale bar = 10 μm. (TIF) [file pbio.3002980.s004.tif]

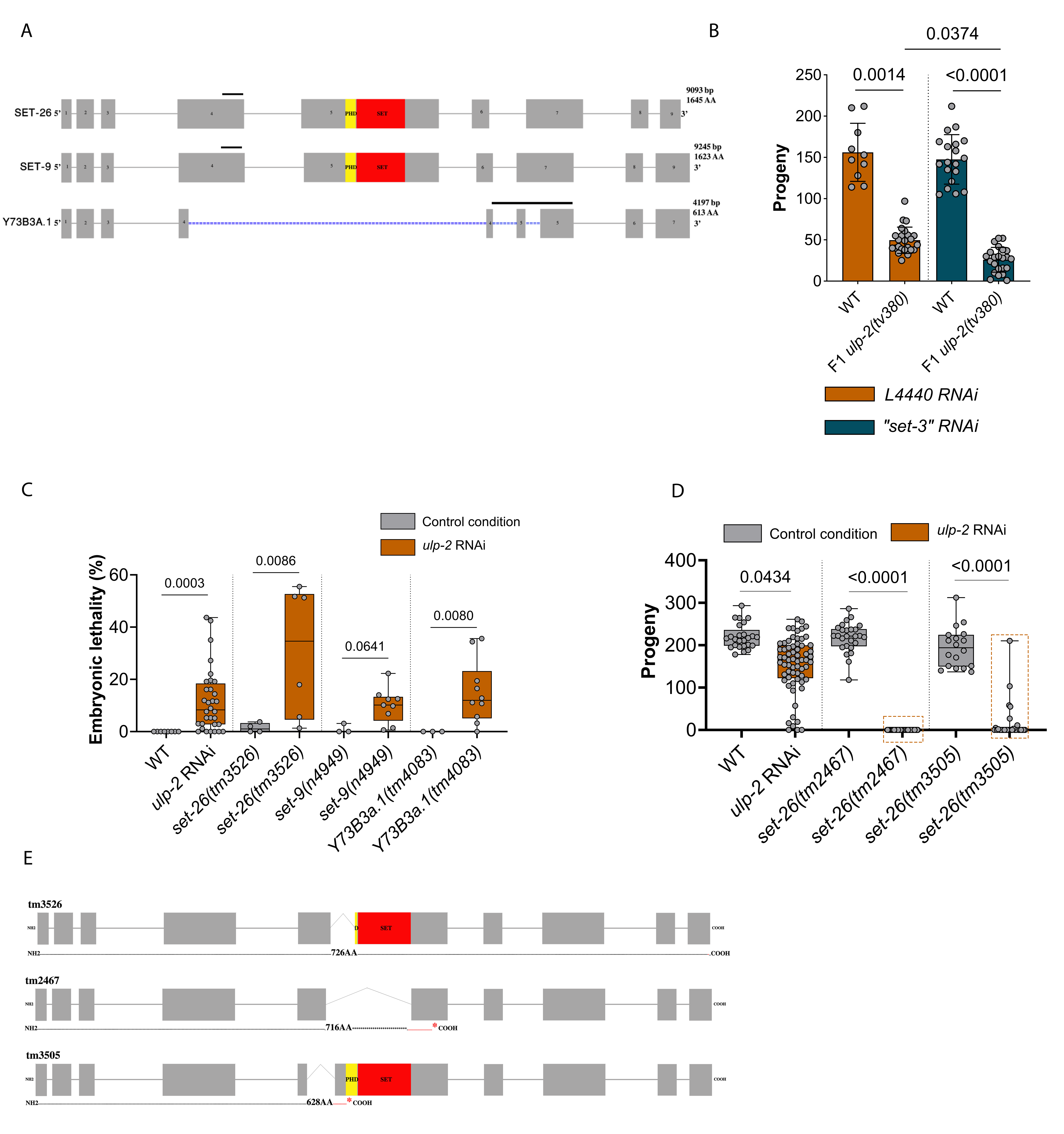

Supplement: S5 Fig — (A) Schematic representation of the exons composing the 3 family members identified in the Y2H screen; SET and PhD domains encoding exons are highlighted in red and yellow, respectively. The sequences including the SET and PHD domains are missing in Y73B3a.1 (dashed blue line). Sequences included in the RNAi vectors for the “Set3” RNAi are labeled with black lines above the scheme of each gene. (B) Quantification of brood size of WT (n = 10 worms in control; n = 19 in “Set3” RNAi) and first generation of ulp-2(tv380) mutant animals (n = 24 in control; n = 23 in “Set3” RNAi); Shapiro–Wilk and one-way ANOVA on ranks (Kruskal–Wallis) followed by Dunn’s post hoc test was used, ns = p > 0.05. (C) Quantification of embryonic lethality of WT (n = 8 worms), ulp-2(RNAi) (n = 32), set-26(tm3526) (n = 4), set-26(tm3526);ulp-2(RNAi) (n = 6), set-9(n4949) (n = 3), set-9(n4949);ulp-2(RNAi) (n = 9), Y73B3a.1(tm4083) (n = 3), and Y73B3a.1(tm4083);ulp-2(RNAi) (n = 10); Shapiro–Wilk and one-way ANOVA on ranks (Kruskal–Wallis) followed by Dunn’s post hoc test was used, ns = p > 0.05. (D) Quantification of the amount of progeny of WT (n = 25 worms), ulp-2(RNAi) (n = 65), set-26(tm2467) (n = 26), set-26(tm2467);ulp-2(RNAi) (n = 80), set-26(tm3505) (n = 18), and set-26(tm3505);ulp-2(RNAi) (n = 30); Shapiro–Wilk and one-way ANOVA on ranks (Kruskal–Wallis) followed by Dunn’s post hoc test was used, ns = p > 0.05. (E) Schematic representation of WT and set-26 deletion alleles (NBRP, Japan) set-26(tm3526), set-26(tm2467), and set-26(tm3505), early stop codon is labeled in red. The numerical data presented in this figure can be found in S1 Data. (TIF) [file pbio.3002980.s005.tif]

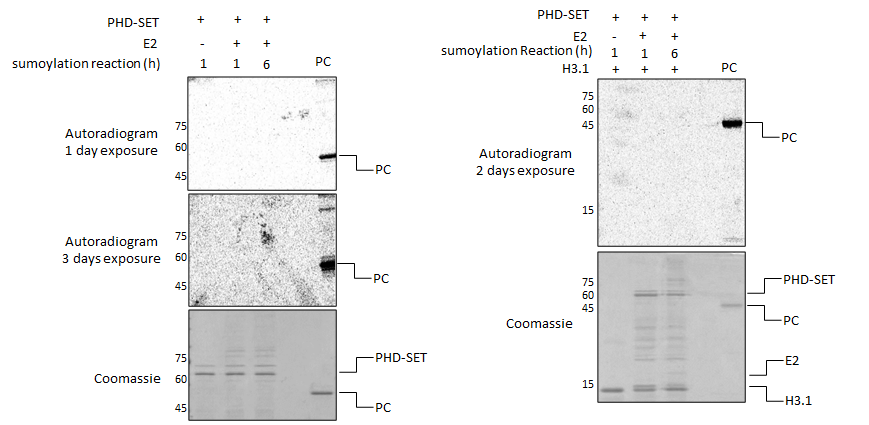

Supplement: S6 Fig — (A, B) Recombinant GST-PHD-SET of SET-26 was sumoylated for the indicated time points with or without the addition of E2 (UBC9). Samples were then subjected to an in vitro methylation reaction in the presence of 3H-labeled SAM without (A) or with recombinant Histone H3.1 (H3.1) as a substrate (B). Samples were subjected to SDS–polyacrylamide gel electrophoresis (PAGE) followed by exposure to autoradiogram as indicated. Human SETD6 served as positive control (PC). Coomassie stain of the recombinant proteins used in the reactions is shown on the bottom. (TIF) [file pbio.3002980.s006.tif]

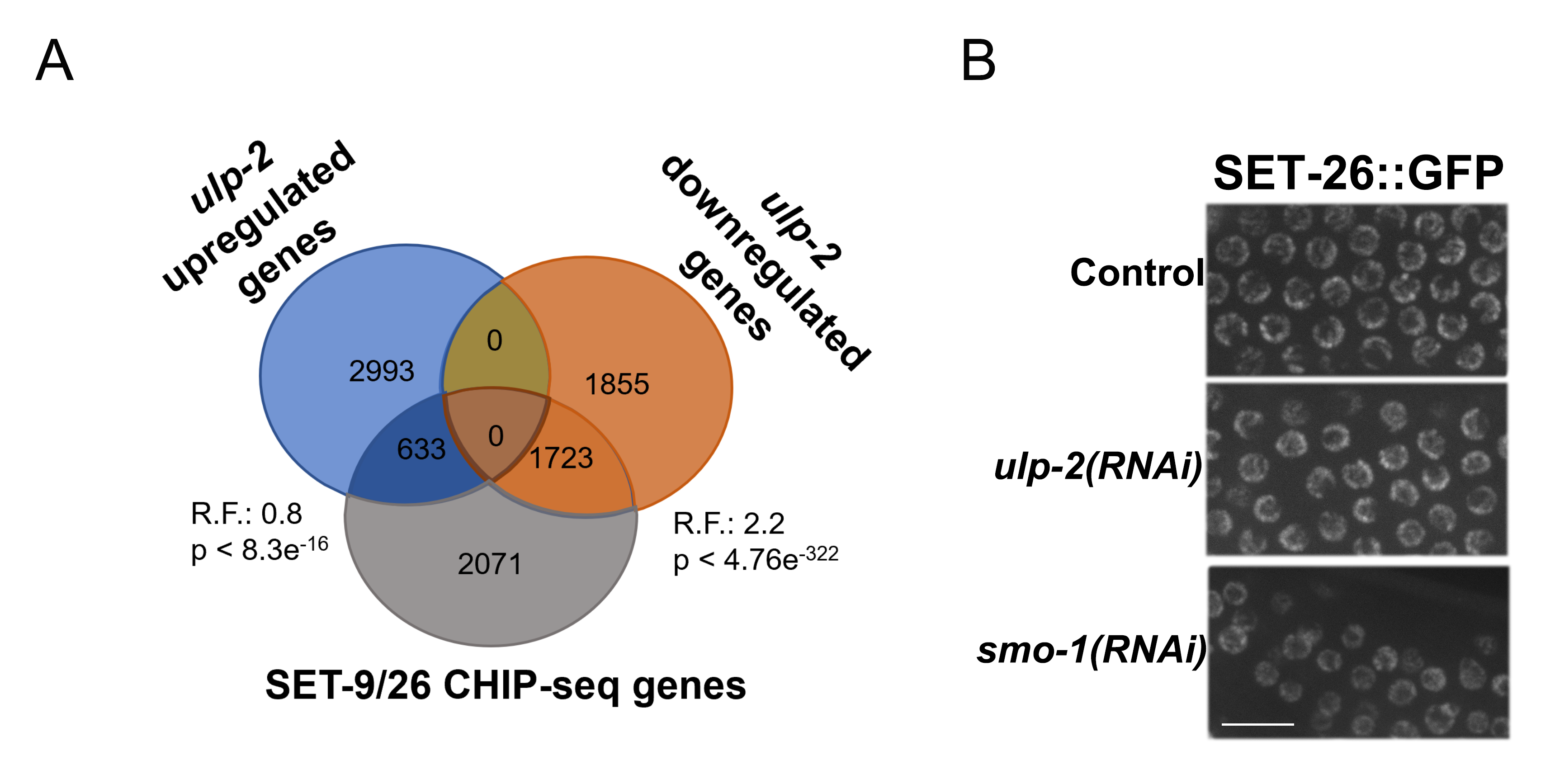

Supplement: S7 Fig — (A) Venn diagram representing intersection between the differential expressed genes in F2 ulp-2(tv380) germlines and the SET-9/26 binding genes (CHIP-seq); Fischer’s exact test was used, ns = p > 0.05, R.F. = Representation factor. (B) SET-26::GFP is localized at the nuclear periphery and within the nucleoplasm. Confocal GFP analysis of set-26::GFP in control conditions (L4440 vector, n = 7); ulp-2(RNAi) (n = 7) and smo-1(RNAi) (n = 7). Scale bar = 10 μm. (TIF) [file pbio.3002980.s007.tif]

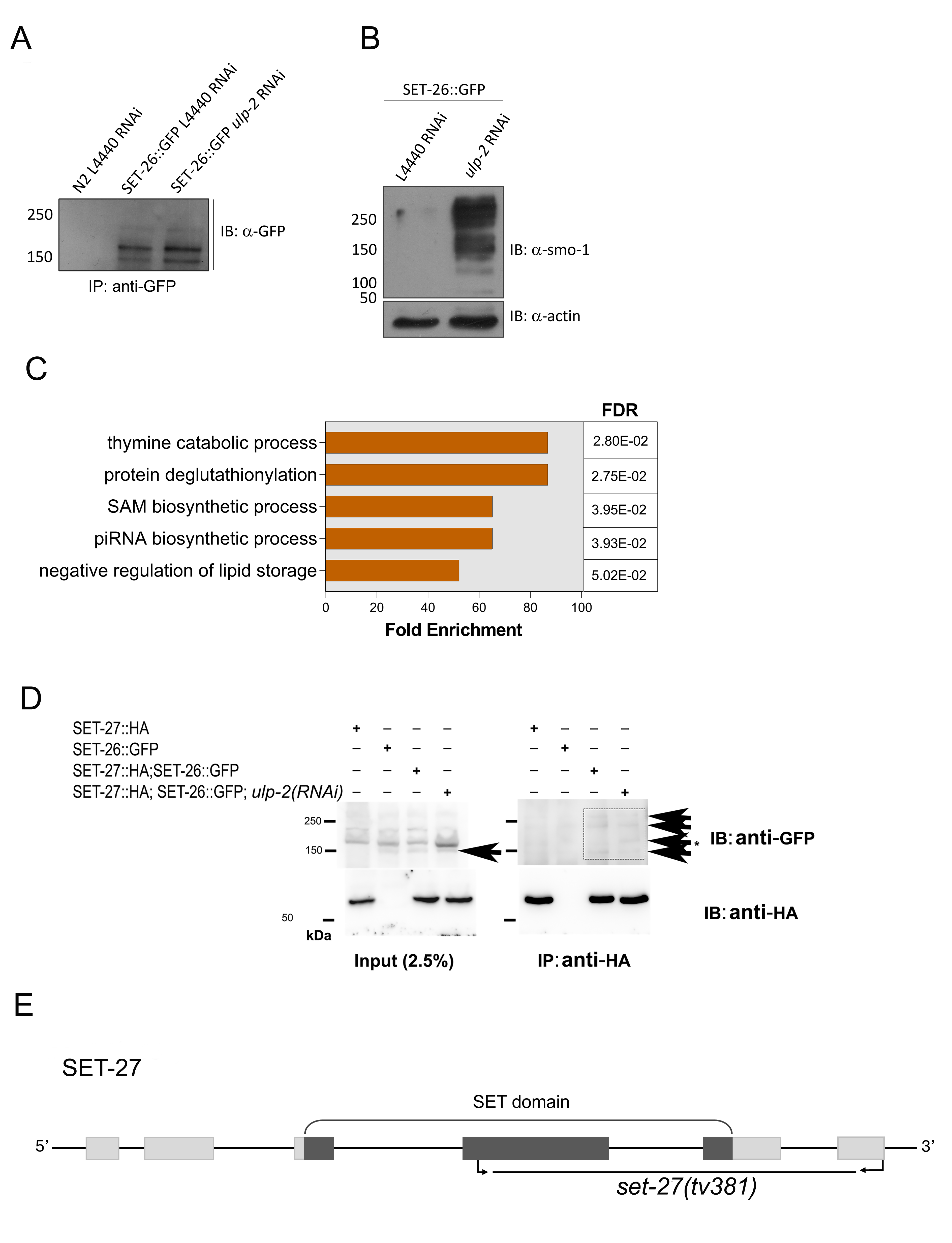

Supplement: S8 Fig — (A) Representative immunoblot of the IP of SET-26::GFP samples sent to Mass Spectrometry; N2 L4440 RNAi was used as a control for GFP immunoprecipitation, SET-26::GFP L4440 RNAi corresponds to SET-26::GFP in a WT background and SET-26::GFP ulp-2(RNAi) corresponds to SET-26::GFP IP in the excessive SUMOylation background; 3 biological replicates. (B) Representative immunoblot showing excessive SUMOylation in the knockdown of ulp-2 by ulp-2(RNAi) when compared to the control (L4440 RNAi). (C) Gene Ontology analysis of the WT interacting partners of SET-26::GFP. Data in S4 Table. (D) Co-immunoprecipitation of SET-27::HA with SET-26::GFP. The bands in the anti-GFP blot appear weaker in ulp-2(RNAi) immunoprecipitation accompanied by the appearance of additional putative isoform-specific SET-26 band (*). (E) Exon intron structure of SET-27 and set-27(tv381) deletion allele. The light gray rectangles and dark rectangles are exons, the dark part of the rectangles is the SET domain, the arrows label the crRNAs binding sites, and the deletion is highlighted. (TIF) [file pbio.3002980.s008.tif]
