# Supplementary material for: SUMO-mediated regulation of H3K4me3 reader SET-26 controls germline development in C. elegans
Source: PLoS Biol. 2025 Jan 6;23(1):e3002980. doi: 10.1371/journal.pbio.3002980 (PMC11703099; doi:10.1371/journal.pbio.3002980)
Supplement: S1 Raw Images — (PDF) [file pbio.3002980.s009.pdf]

A

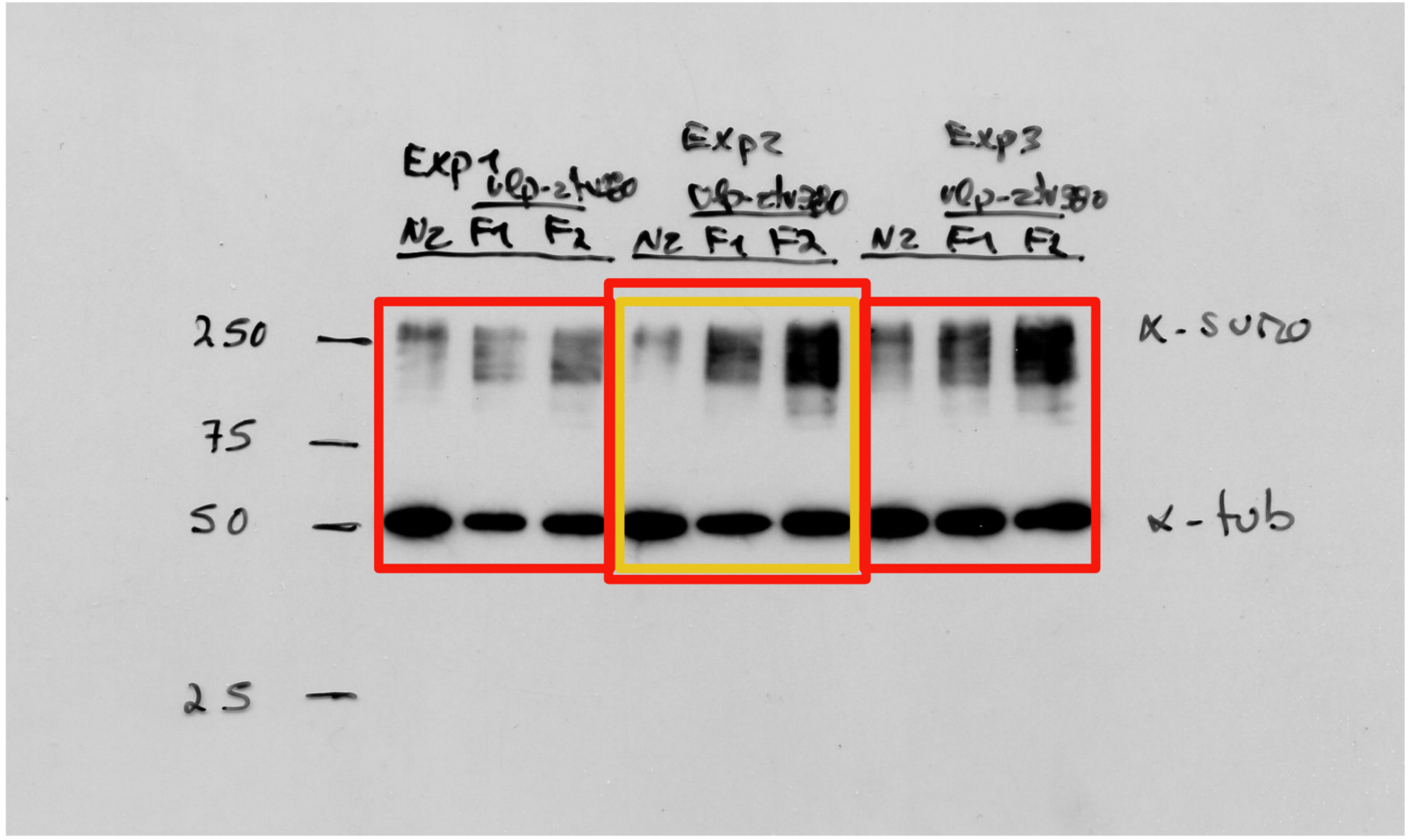

B

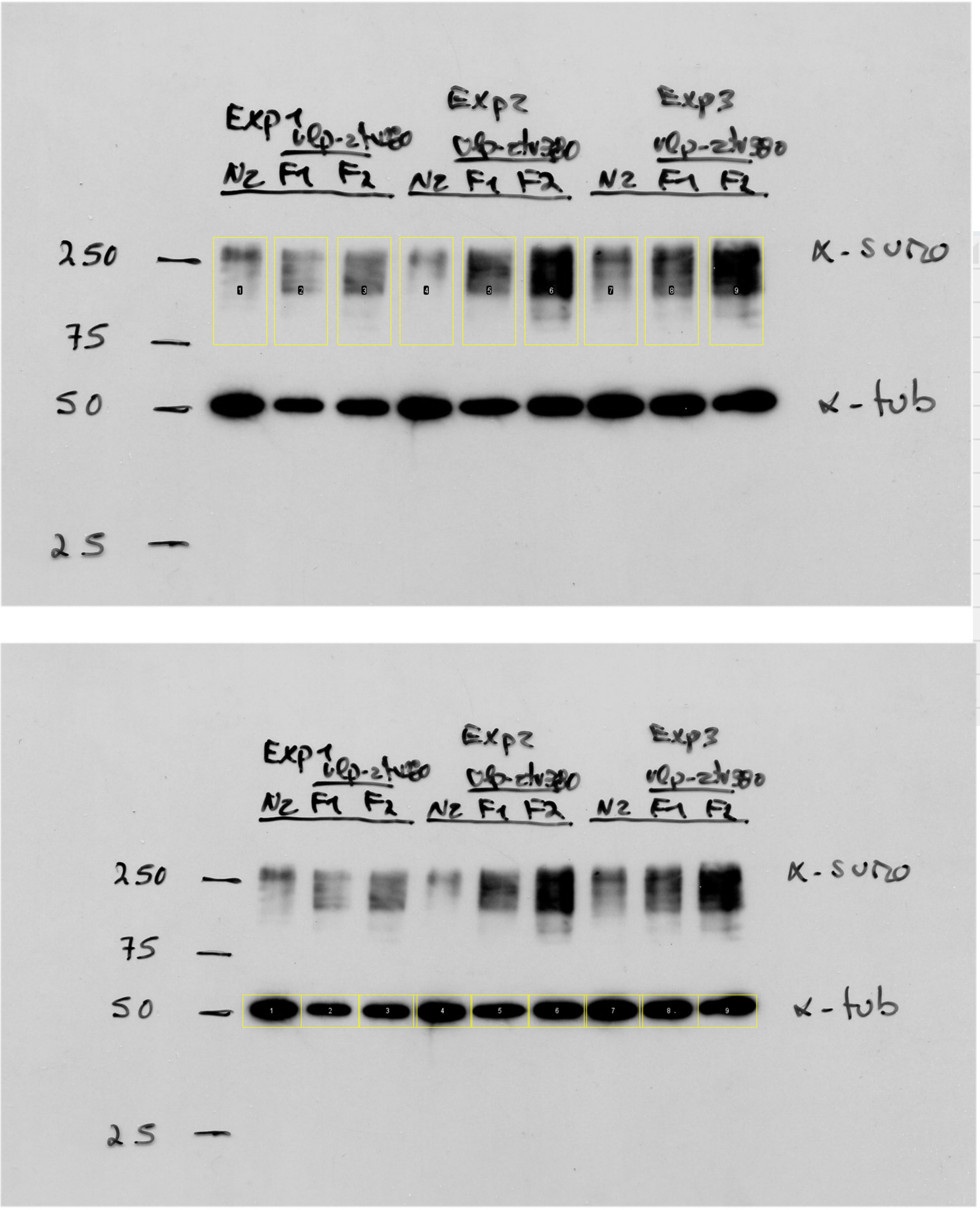

area measurements of peaks of WB for total sumo levels in N2 vs F1 ulp-2tv380 vs F2 Ulp-2tv380

Genotype	peak area "total	peak area "tubulin"	Normalization "total sumo"/"tubulin"
N2 1	20111.25	31074.735	0.647189751
F1 Ulp-2tv380 1	21434.128	18531.43	1.156636482
F2 Ulp-2tv380 1	34563.978	21055.108	1.641595854
N2 2	13994.229	31678.806	0.441753676
F1 Ulp-2tv380 2	48601.011	25230.643	1.92626922
F2 Ulp-2tv380 2	85352.96	30483.907	2.799935061
N2 3	33136.798	35176.584	0.94201296
F1 Ulp-2tv380 3	52464.375	32096.735	1.634570463
F2 Ulp-2tv380 3	89628.295	31501.179	2.845236205

Figure 1D. A. scans **B.** quantification

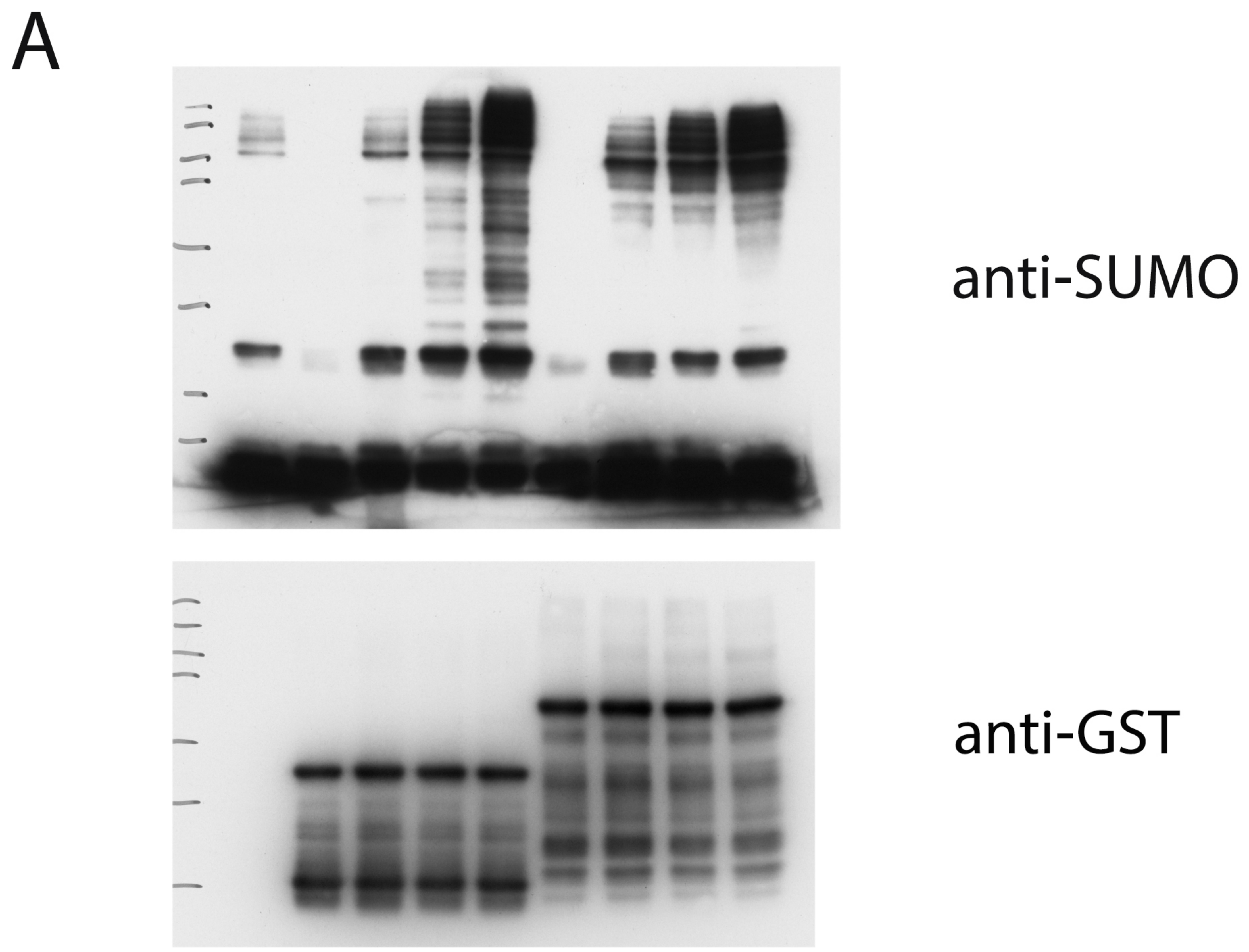

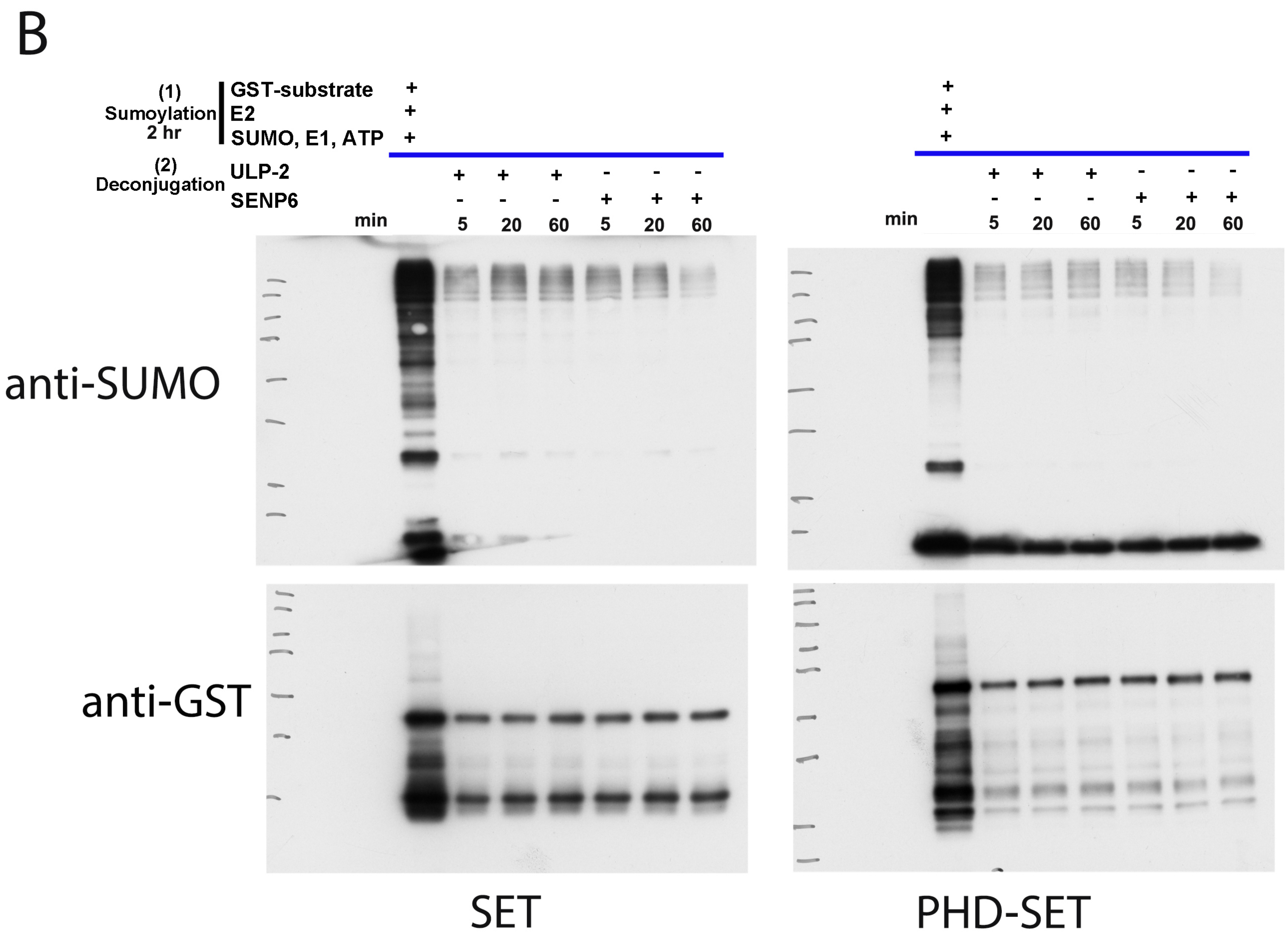

Scans Figures 4A-B

A. Scans Figure 4A **B.** Scans Figure 4B

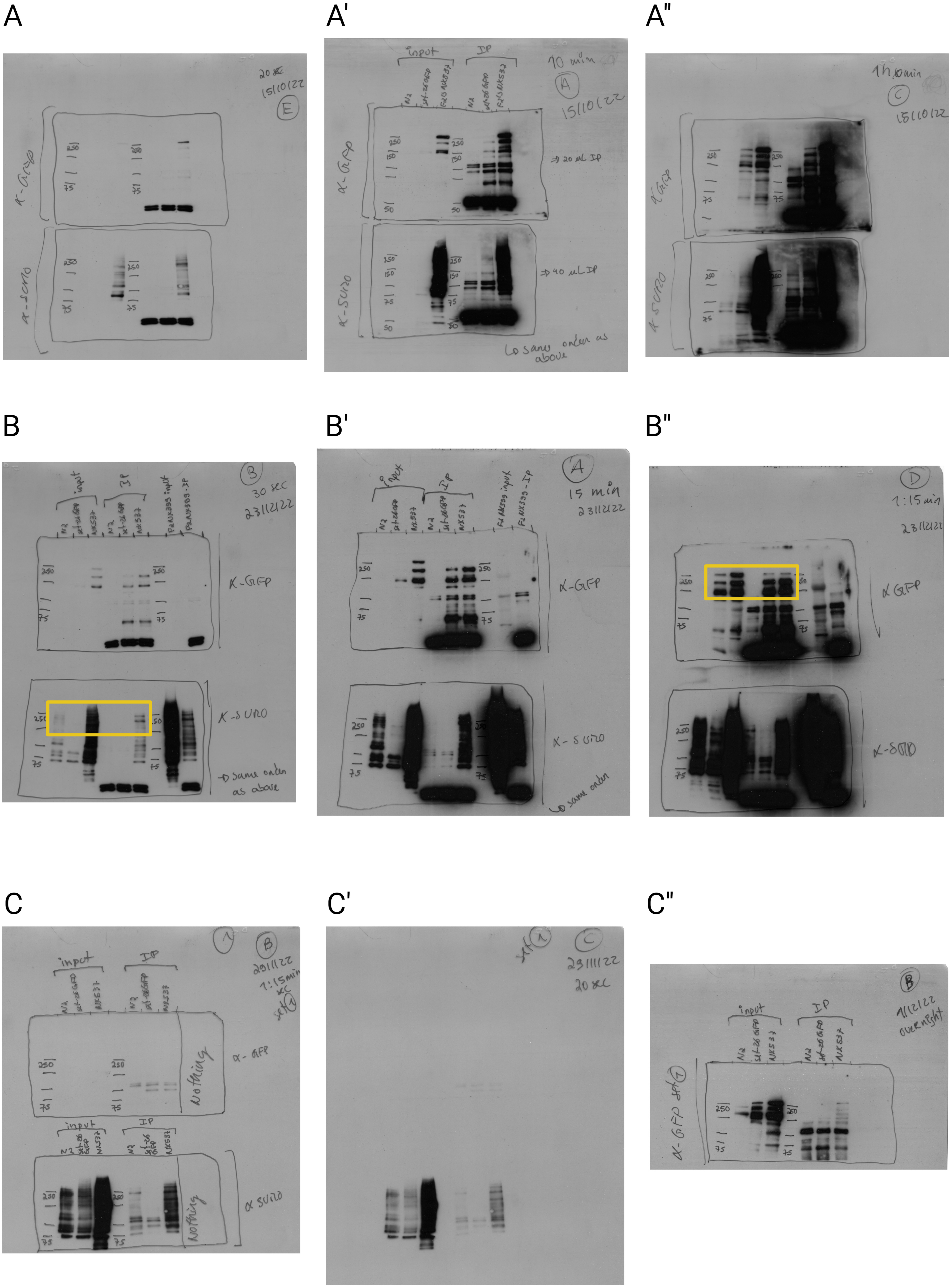

Figure 4C. scans of the three experimntal repeats A,B,C

Exposures used for quantification (Figure 4D)：A and A', B and B', C'and C "

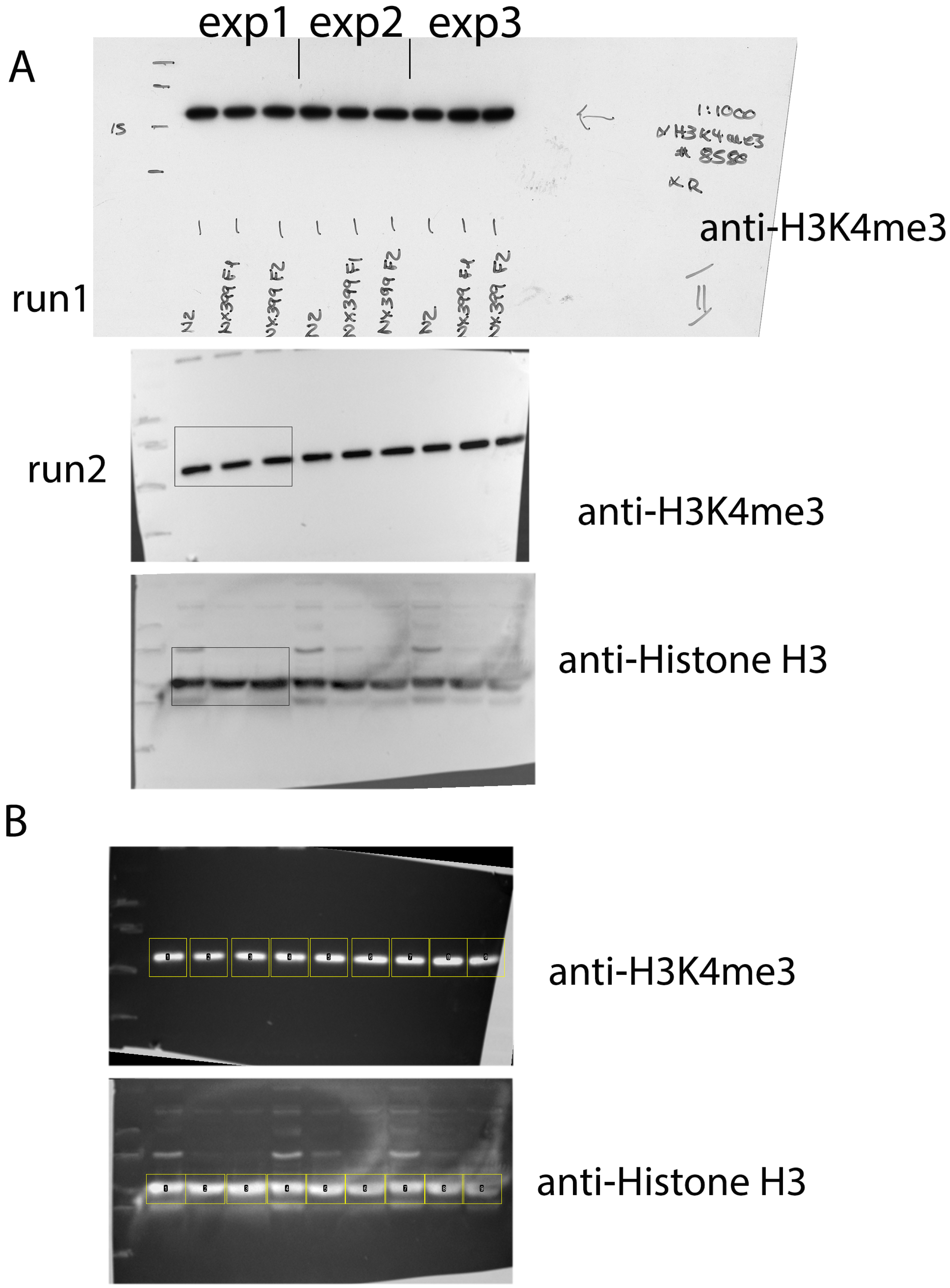

samples	peak numt	H3 total ar	H3K4me3		H3K4me3/H3 total
N2 1	1	24351.4	12851.63		0.527757356
F1 ulp-2tv3	2	23394.52	11187.27		0.478200281
F2 ulp-2tv3	3	26682.89	12911.46		0.483885401
N2 2	4	19775.89	13376.41		0.676399985
F1 ulp-2tv3	5	18888.06	12875.39		0.68166821
F2 ulp-2tv3	6	17183.89	14583.46		0.848670214
N2 3	7	14173.43	13037.63		0.919864211
F1 ulp-2tv3	8	18168.21	14156.51		0.779191321
F2 ulp-2tv3	9	14464.49	11594.17		0.801560774

Figure 5A. scans of the three experimntal repeats

A. scans

B. quantification

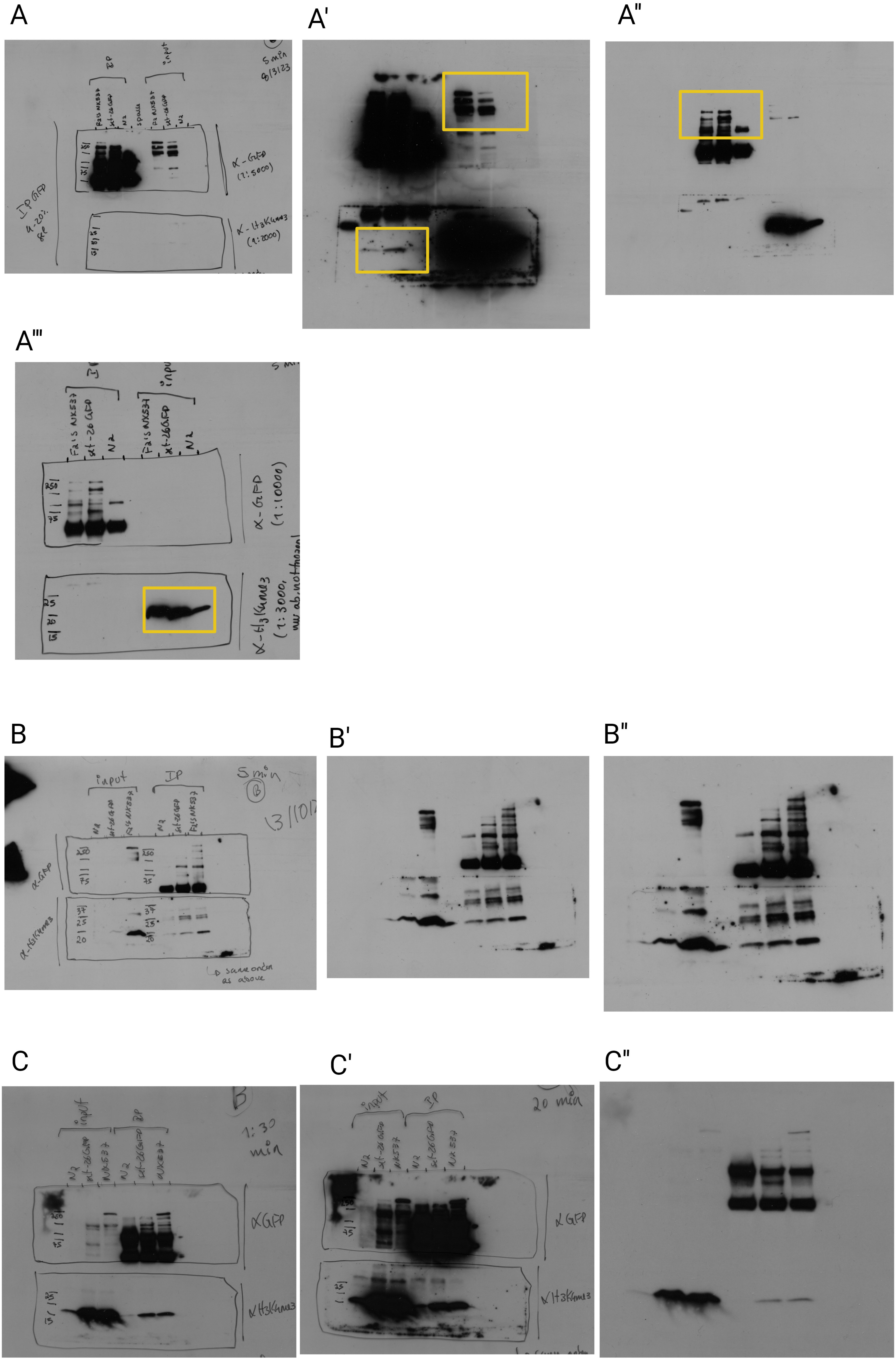

Figure 5B. scans of the three experimntal repeat
A′ and A″, B′ and C were used for quantification

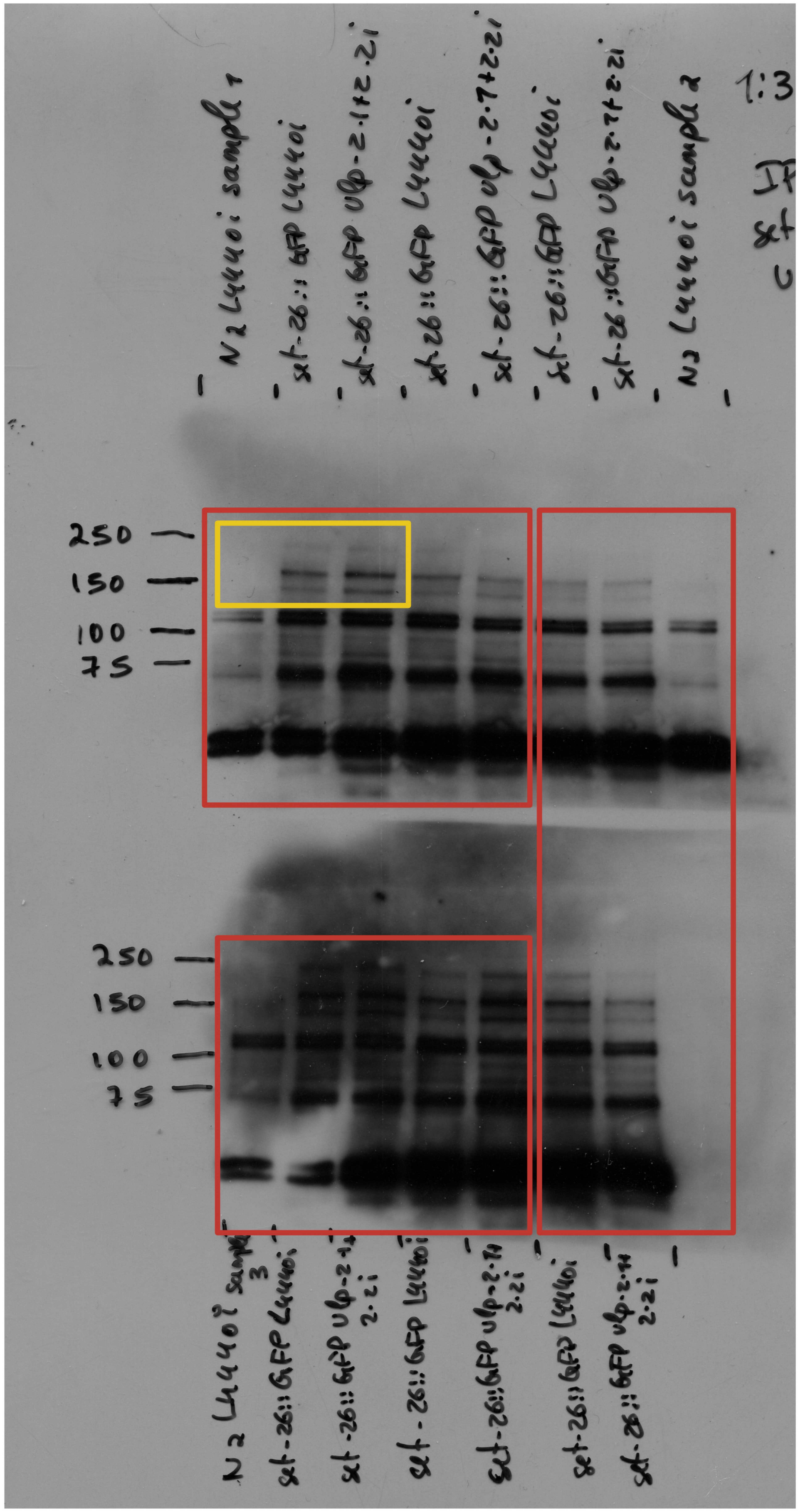

Figure S8A. scans
Qualitative verification of set-26 presence for MS

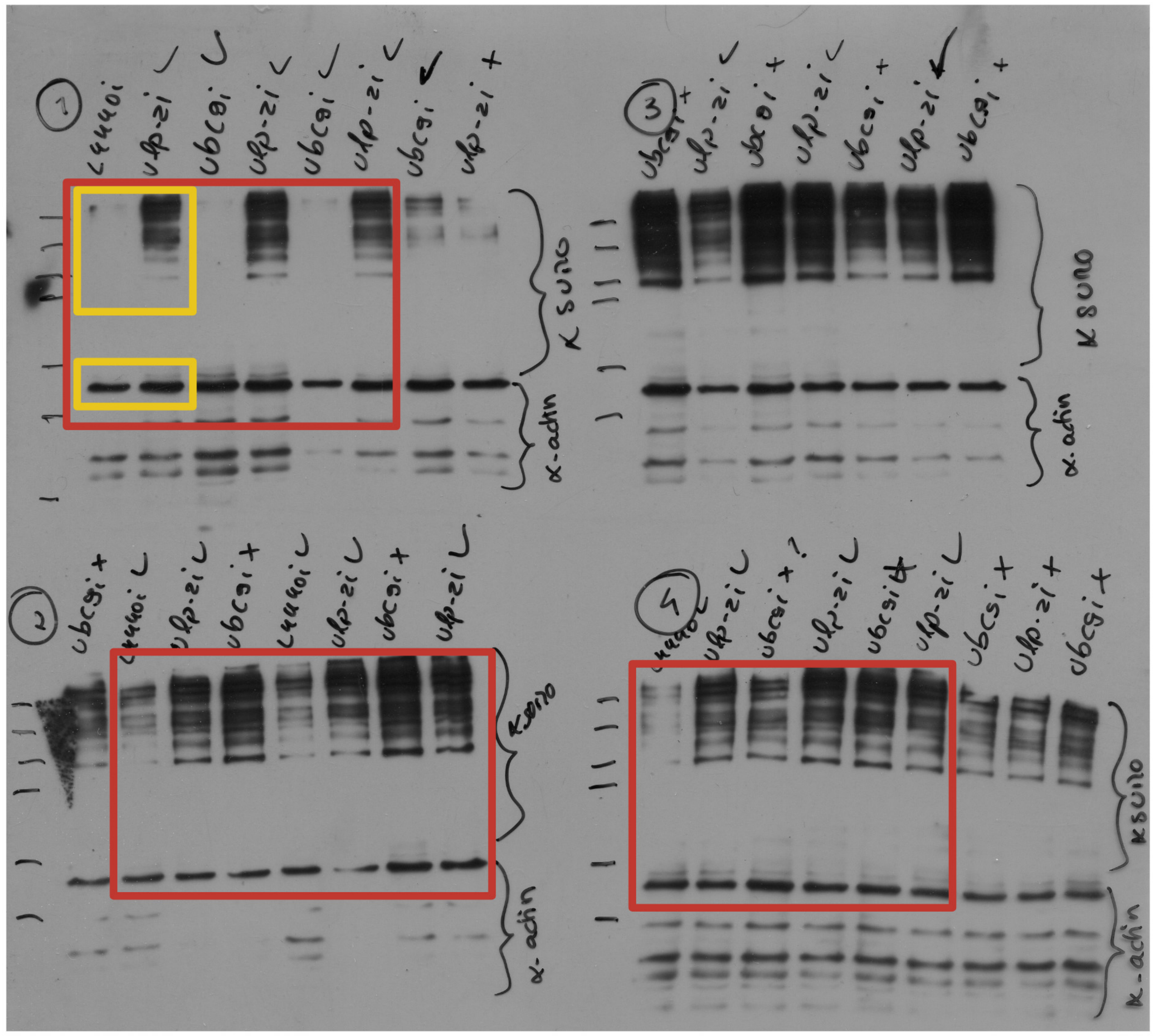

Figure S8B. scans
Qualitative verification of RNAi efficacy in SET-26::GFP for MS

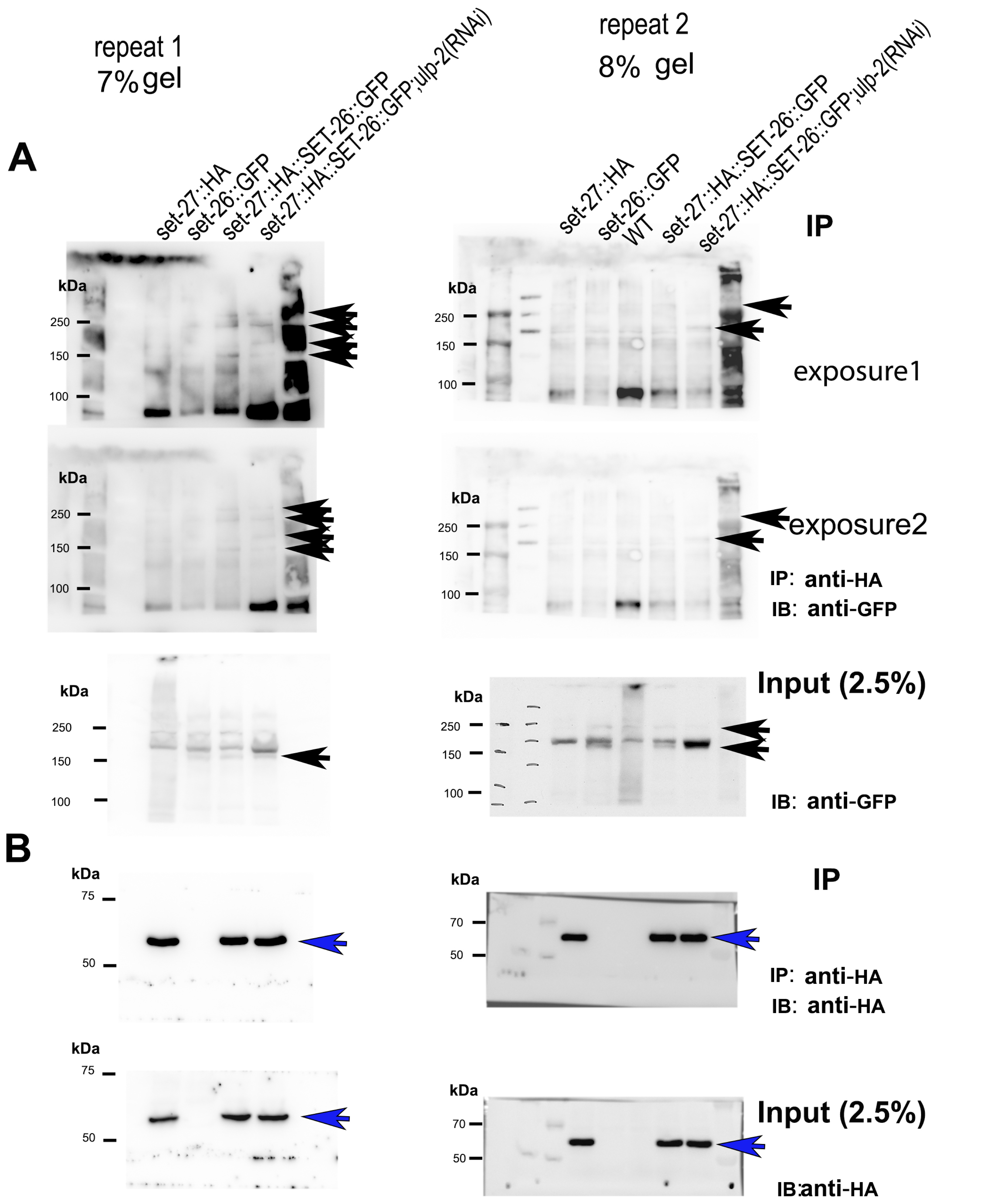

Figure S8D. scans of the three experimntal repeats

Immunoprecipiaiton with anti-HA

A. anti-GFP

B. anti-HA
