# Supplementary material for: SUMO-mediated regulation of H3K4me3 reader SET-26 controls germline development in C. elegans
Source: PLoS Biol. 2025 Jan 6;23(1):e3002980. doi: 10.1371/journal.pbio.3002980 (PMC11703099; doi:10.1371/journal.pbio.3002980)
Supplement: Attachment — Submitted filename: resposne to reviewers _plosbio_270924.pdf [file pbio.3002980.s017.pdf]

RESPONSE TO REVIEWERS

PBIOLOGY-D-24-00826R1

SUMO-mediated regulation of a H3K4me3 reader controls germline development in *C. elegans*

EDITORIAL REQUESTS

The reviews are attached below. As you will see, the reviewers find the conclusions interesting and significant, but they also raise several concerns that would need to be addressed before we can consider the manuscript for publication. Reviewer 1 thinks you should perform additional experiments to measure growth rates, to check if knocking down SMO-1 can rescue the offspring number phenotype or SUMO accumulation, and to validate the interaction between SET-26 and SET-27. Reviewer 2 would like you to confirm the germline to soma transformation by using endogenous markers and also to rewrite the paper and improve the figures to make it is more accessible for a broad audience.

We have completed the following main experiments:

1. Recorded the development of WT and *ulp-2* mutant animals to compare the growth rates from L1 to adulthood in 12 hr intervals at 20°C. (Figure S1)

2. Performed *smo-1(RNAi)* to check if can rescue the *ulp-2* mutant animals (Figure in PBP).

3. Analyzed SET-26::GFP expression pattern under *ulp-2* or *smo-1* downregulation (Figure S7).

3. Validated the physical interaction between SET-26 and SET-27 (Figure S8).

4. Used a crispr-tagged reporter to examine the loss of germline fate (Figure S4).

5. Modified the manuscript and figures as suggested by the reviewers.

We responded to all the points raised by the reviewers below:

POINT-BY-POINT RESPONSE

We thank the reviewers for their valuable comments and suggestions, which were most helpful in revising and improving our manuscript. Below is a detailed point-by-point response to each of the comments.

Reviewer #1

The work focuses on the role of ULP-2, a SUMO protease, in controlling germline development in C. elegans. It highlights ULP-2's regulation of SET-26, a PHD-SET domain protein. The study found that ULP-2 mutant showed increased sterility and altered global protein SUMOylation. RNAseq analysis revealed gene expression changes in ulp-2 mutant gonads, indicating a loss of germline gene expression and increased somatic gene expression. The paper also discussed how SET-26's SUMOylation is regulated by ULP-2 and the impact on H3K4me3 binding. This work presents intriguing discoveries in the post-translational modification of histone readers, exhibits commendable innovation and delves into the mechanisms of epigenetic regulatory mechanisms. Overall, this is an important discovery in the field of epigenetic regulation, yet need major revision before publication. Especially, some areas need refinement, and certain conclusions require further evidence.

1. Regarding the statement, "These results reflect a progressive increase in the lifespan of animals lacking ULP-2 activity, which correlates inversely with its reproductive function,". An additional experiment measuring growth rates could provide more insights.

We performed a growth rate experiment of WT and mutant animals. The results appear in a new Supplementary Figure S1 (with the numerical data in S1 Data). The data shows that *ulp-2* mutant animals develop more slowly compared to WT animals as shown in Figure 1A, therefore the growth rate correlates with ULP-2 activity and reproduction and inversely correlates with lifespan. Important to note is that lifespan measurements start at day 1 of adulthood.

2. It would be interesting to see if knocking down SMO-1 can rescue the offspring number phenotype or SUMO accumulation. Exploring whether knocking down SMO-1 changes the protein-protein interaction outcomes dependent on ULP-2 for SET-26 would add depth to the study.

Analysis of *smo-1(RNAi)* did not rescue the offspring numbers of the *ulp-2* mutant animals but instead, enhanced the sterile phenotype. While the *smo-1(RNAi)* led to F2 embryonic lethality (black arrows) (Jones et al, Genome Biology 2002, PMID: 11806825), it caused complete sterility of the F1 animals when done in an *ulp-2(tv380)* background. The image shows a representative plate from each condition (n=30 for each condition, single F1 homozygous or WT at the L3 stage was plated and the number of progeny were counted).

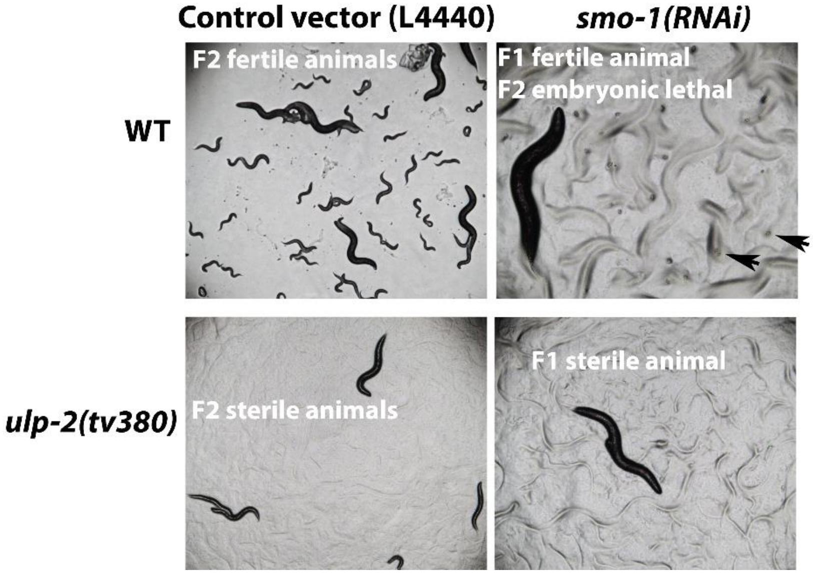

This observation prevented our ability to measure the offspring number and SUMO accumulation levels. It is also important to note that both the single SUMO peptide and the four non-redundant SUMO proteases in *C. elegans* have multiple targets with various dynamics of the modification and therefore the enhanced sterility suggests that the activity of additional proteins required for fertility were impaired due to the inhibition of their sumoylation. Regarding the effect of downregulation of SUMO on the protein-protein interactions of ULP-2 and SET-26: we thank the reviewer for this comment, this will probably allow the discovery of additional components of ULP-2 and SET-26 dynamic complex and will be part of a future study. Due to the lethality we observed we will need to build a system of conditional knockdown.

3. Additionally, it would be informative to examine whether and how ulp-2 and SUMO impact the expression and localization of SET-26.

We performed analysis of SET-26::GFP localization in the germline using super-resolution spinning disk confocal microscopy following *ulp-2(RNAi)* or *smo-1(RNAi)* and control (empty vector). The data is presented in a new Supplementary Figure S7. We found that SET-26::GFP is expressed at the nuclear periphery and within the nucleoplasm. Although we detected the typical defects in the germlines of the RNAi animals (abnormal germlines with less nuclei in both *ulp-2* and *smo-1* RNAi treated animals and smaller nuclei in *smo-1(RNAi)*), we could not detect changes in the localization pattern between the different conditions. We conclude that impaired sumoylation and desumoylation levels by downregulation of *ulp-2* and *smo-1* do not disrupt SET-26 localization in the germline.

4. Fig 5: The total amount SET-26::GFP is much higher in ulp-2;set-26::gfp than in set-26::gfp, but the IPed set-26::gfp in ulp-2;set-26::gfp is obviously lower than from set-26::gfp sample. Are these representative images.

Additionally, the reason to choose H3K4me3, but not include other histone modification markers, should be clarified? What are the genomic loci with changed H3K4me?

These are representative images and the original films and repeats used for quantification are presented in S1_raw images. It was technically challenging to immunoprecipitate the SET-26 protein which showed sensitivity to many parameters, for example, extraction buffer, stability at 4ºC, antibody batch, strain differences as well as minor changes in the growth conditions of the animals that we cannot always control. For this reason, the amounts we isolated in the extract and detected as the input and the amounts detected after IP from the same extract are not comparable. Moreover, the amounts of IPed protein varied between the IP reactions. Although we tried different strategies and performed multiple attempts, we could not optimize this IP. We therefore performed the quantifications relative to the amount of SET-26 we could detect in each IP.
We focused in this study on H3K4me3 modification because it is the modification that SET-26 is known to bind to as an H3K4me3 reader protein (PMID: 29714684). Please see also comment 6/6 below. The genomic loci with changed H3K4me3 are most probably the SET-26 bound loci that intersect with the differential expressed genes in *ulp-2(tv380)* and shown in Figure S7A.

5. Fig 6: Additional evidence to support the interaction between SET-26 with SET-27 and its regulatory significance is needed. A second assay, either in vivo or in vitro, could strengthen this claim by confirming that ulp-2 RNAi indeed leads to weakened protein-protein interactions.

We labeled SET-27 endogenously with HA-tag and performed immunoprecipitation to SET-27::HA;SET-26::GFP double labeled strain to confirm the mass spectrometry results. The results are presented in Figure S8D. Overall the bands are weak but specific. As detailed in comment (4) above it is difficult to IP SET-26. We have also tried the IP with GFP but there was a background band in the MW size of SET-27. Regarding *ulp-2(RNAi)*: as shown in Figure S8D, the bands were weaker in the IP of the *ulp-2(RNAi)* extract although a band corresponding to a putative specific isoform of *set-26* appeared only in the immunoprecipitation of the *ulp-2(RNAi)* extract.

6. Minor:

1. Fig 1D: Please clarify why the molecular weight of sumoylated proteome ranges from 100-250 kD but not lower.

We cut the membrane after the transfer, the upper part was used to blot with anti-SUMO and the lower part was used to blot with anti-tubulin. In addition, we indeed observed that in our hands, this antibody binds with some higher affinity to higher molecular weight SUMO-conjugates as visible in the WT control. When the amount of SUMO-conjugates increases or when the blots were exposed for a longer time we could also detect the lower molecular weight conjugates, for example please see the original scans of Figure 4C in the input lanes (panels B on the left side) in S1_raw images.

2. Fig 1E: in panel E, can't understand how the signals were quantified and compared. According to panel D, the differences are so obvious.

We added in S1_raw images file, in the scans of Figure 1D the quantification lanes saved from Fiji and the quantification table. Each lane was quantified using the same defined area. After that, the pixels intensities were plotted and the area below each curve measured. This value was divided by the tubulin value of each lane. One of the three biological repeats scored lower and decreased the average down.

3. Fig 2C: The labeling in this panel is not clear. Please revise for better clarity.

We re-labeled the panel and enlarged the image. We added dashed circle around a nucleus in diplotene, square around a nucleus in diakinesis, arrow on a possible somatic nuclei.

4. Fig 2E: Does soma no longer express unc-119::GFP in F2 ulp-2(tv380)? This needs to be addressed.

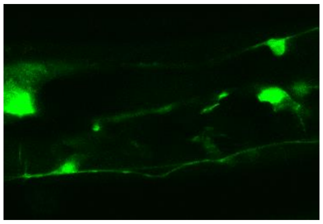

This reporter is pan-neural. The neuronal expression was not changed in *ulp-2* mutant animals. The focal planes of the ventral and dorsal nerve cords that can be detected in the gonad region are below or above the gonad. This is an image from a different focal plane of the same animal showing GFP expression in some of the neuronal processes.
Following this comment, we noticed that the graphs in Figure 2F were not correct because all animals expressed this reporter in the neurons but not all of them showed ectopic germline expression and this is what we meant to quantify. In the revised manuscript we deleted this graph and detailed the quantification in the result section in line 198. In addition, following the request of reviewer #2 we also added the analysis of an additional reporter shown in Figure S4.

5. Fig 2B: It is very likely that ulp-2 primarily affects the proteome, thereby indirectly impacting the transcriptome? Therefore, including a transcriptome assay does not necessary reflect the function of ulp-2 in vivo.

SUMO is a major regulator of nuclear functions, including the regulation of many transcription factors. Based on the sterile phenotype we predicted that abnormal sumoylation dynamics due to loss of *ulp-2* impairs the activity of germline transcription factors. To reveal directly what are the key changes in germline gene expression we decided to start with RNAseq of dissected gonads that indeed enabled us to detect the changes in the full landscape of germline transcription. Proteomics of dissected gonads is still a technical challenging task for lowly expressed proteins, however we do plan to perform this experiment in the future when more advanced proteomics approaches become available.

6. Fig 2G: The reasoning for choosing H3K27me3 over other markers like H3K9me3 is not apparent. Also, a quantification of the Western Blot results is suggested.

We agree with this comment and removed this data from the manuscript. We focus on H3K4me3 due to the interaction with the H3K4me3 reader, SET-26. Accordingly, Figure 2G appear now in Figure 5A.

7. Fig 3A: What are the control pairs in the Y2H assay? Are they the same two proteins or different known interactions? If same two proteins, how reproducible is this assay. The range of set-26 N & C should be indicated within a schematic domain structure of SET-26.

We added in the methods section in line 564: "Control pairs express various AD/DB fusion pairs (see Table S3) that result in known yeast growth characteristics on the different assay plates, from no growth to strong growth, and act as a benchmark to gauge the strength of Y2H interactions". The table below was added in Supplementary Table S3 (S3_table). The numbers: 0 is defined as no growth, 1 as weak growth, 2 as medium growth, and 3 as strong growth (equal to no selection condition).

Number	DB clone	AD clone	-Leu -Trp -His	-Leu -Trp -His + 2 mM 3AT	-Leu -Trp -Ade	-Leu -His +Cycloheximide
1	pPC97 -Cyclo (empty vector)	pPC86 (empty vector)	0	0	0	0
2	dpm-1	fars-1	2	1	0	3
3	mbk-1	Y79H2A.3a (aa468 – 710)	3	2	0	0
4	pas-6	pas-7	3	3	0	0
5	rfc-3	eif-3.D	3	3	1	3
6	Fos HLH domain	Jun HLH domain	3	3	2	3
7	DP1	E2F1 (aa157-436)	3	3	3	3

In addition, the range of set-26 N & C is now indicated in Figure 3B.

8. Fig 3B: are tm2467 or tm3505 nulls? Is the interaction domain between set-26 & ulp-2 important? Does set-26 show progressive sterility as ulp-2?

We have not tested if these alleles of *set-26* are genetic nulls. As far as I know it was not tested in other publications on SET-26. Because both these alleles lack the PHD-SET domain we can be confident that they lost their intrinsic H3K4me3 reader ability. However, we do not know if another readers in their vicinity can complement their activity. We have not tested the functional role of the interaction domains of SET-26 and ULP-2. We report the interaction domain here to provide another support for the specific physical interaction between these proteins. According to the data published in PMID: 29714684 from the Lee lab, SET-26 does not show progressive sterility, however, double mutants of *set-26;set-9* exhibit progressive sterility starting from the F2 generation
https://www.ncbi.nlm.nih.gov/pmc/articles/PMC6010342/figure/fig2/
https://www.ncbi.nlm.nih.gov/pmc/articles/PMC6010342/figure/fig2s1/

9. Fig 3C: It is very hard to recognize the genotype and lifespan based on so many colors. Need include an additional column chart to compare the media lifespan.

The data was divided into two graphs and appears in Figure 1C and Figure 3C. A summary table including median lifespan and statistics for all genotypes is found in Supplementary file S1 Data.

10. Fig 4A: Why does the control lane show a SUMO signal without substrate?

The control lane contains E1, E2, SUMO and ATP. It has been established that Ubc9 can be modified by SUMO (PMID: 18691969), the reaction is less efficient compared to SET-26 sumoylation and was incubated for 60 minutes to detect the weak Ubc9 signal.

11. Fig. 4B: Consider reducing the amount of ULP-2 if the reaction is not in the linear range.

We have tried to achieve this but our attempts failed with ULP-2. We achieved complete desumoylation with human SENP6 that we purified in the same conditions and protocol (left panels). It suggests that the *C. elegans* ULP-2 may need additional cofactors.

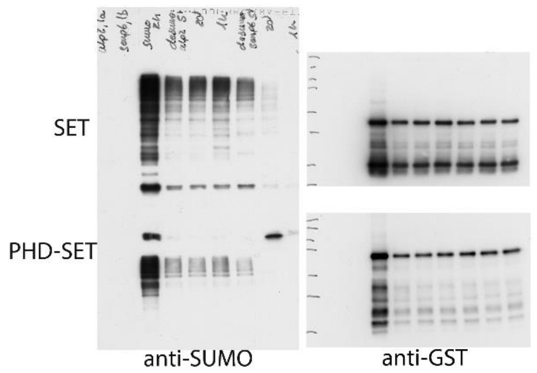

Scans of in vitro reaction comparing ULP-2 and SENP6 desumoylation kinetics

12. Fig. S1C, S3A, and S3F need clearer labeling and visualization.

These figures were corrected including better labeling and visualization. The updated numbers of these figures in this revision:

Original figure	Revised figure
S1C	S2C
S3A	S5A
S3F	S5F

13. Fig. S4: the PC lane has no substrate H3.1, why show up? The two panels should on the same gel. It is an in vitro experiment with bacterial expressed proteins, it is hard to tell in vivo situations, for example, need a cofactor. Or the bacteria expressed protein is miss folded?

In this in-vitro experiment we used SETD6 (PC, positive control) to detect an automethylation activity that serves as an indication for the enzymatic activity. The PC was used to show that the tritium labeled SAM which was added to the reaction can incorporate a methyl group. H3.1 does not serve as a SETD6 substrate and it was placed in the right gel for a positive control as these were independent experiments loaded on two gels. We agree with the reviewer about the in vitro conditions lacking cofactors or generating misfolded proteins. It is accepted that SET-26 is enzymatic inactive based on its homology to MLL5/UpSET/SETD3 (lack important residues required for the methylating enzymatic activity of SET domain proteins) and the in vivo evidence presented in Figure 6 of Wang et al; PMID: 29714684 that was cited in our manuscript. Our question here was to examine if SET-26 can be (transiently) activated following sumoylation and we chose this established in vitro system that has been demonstrated by the Levy group to predict the in vivo activity for multiple SET proteins. References:

1. Elisha L, Elbaz T, Chopra A, Shapira G, Bedford MT, Fry CJ, Shomron N, Biggar K, Feldman M, Levy D: TWIST1 methylation by SETD6 selectively antagonizes LINC-PINT expression in glioma. Nucleic Acids Research 50:6903, 2022.
2. Vershinin Z, Feldman M, Werner T, Weil LE, Kublanovsky M, Abaev-Schneiderman E, Sklarz M, Lam EYN, Alasad K, Picaud S, Rotblat B, McAdam RA, Chalifa-Caspi V, Bantscheff M, Champman T, Lewis HD, Filippakopuolos P, Dawson MA, Grandi P, Prinjha RK, Levy D: BRD4 methylation by the methyltransferase SETD6 regulates selective transcription to control mRNA translation. Science Advances 7:eabf5374, 2021.
3. Feldman M, Vershinin Z, Goliand I, Elia N, Levy D: The methyltransferase SETD6 regulates Mitotic progression through PLK1 methylation. Proc. Natl. Acad. Sci. USA. 116:1235-1240, 2019.

14. Fig. S5D: SET-26 is very likely enriched in the nucleus, but these classes of protein are very likely enriched in cytoplasm?

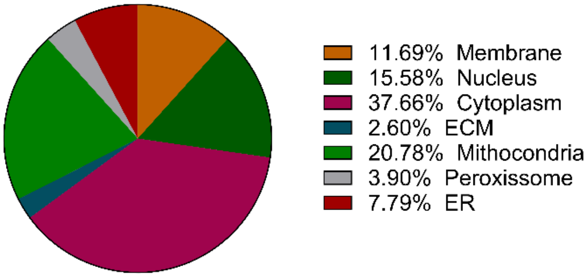

As shown in this pie chart, we analyzed the subcellular localization of the proteins identified in the mass spectrometry analysis presented in Figure 6B. Indeed there are less than 16% nuclear proteins and we suggest few possible explanations. First, the high resolution GFP analysis (Figure S7B) shows dual localization of SET-26 both inside the nucleus and on the nuclear envelope and possibly nuclear pores. This may explain some of the cytoplasmic binding. In addition, these results could indicate on a technical obstacle. To immunoprecipitate SET-26 we had to use tissue fixation with PFA to stabilize SET-26 before pulldown. It is possible that some of these proteins, rather than real interactors, are part of the vicinity of SET-26 that was fixed with PFA and therefore got pulled down with SET-26. In order to know which interactor do functionally interact with SET-26 we will further test them in future research.

Reviewer #2

This manuscript discovers a role for the sumo protease ULP-2 in germline development and potential cofactors. The authors i) define the germline and longevity phenotypes of ulp-2 mutant adults; and ii) establish a physical and functional relationship between ULP-2 and SET-26/MLL5/UpSET. They show that SET-26 is overly sumoylated in ulp-2 mutants and this correlates with weak complex formation with its binding partners and H3K4me association. The implication is that these interactions are critical to repress somatic genes and enhance germline expression in the germ line. The authors have undertaken a range of methods to analyze ulp-2 and define its germ line role.

General comment
The paper would be improved and probably garner more readership, if the authors could give more biological context to their findings in the Abstract, Introduction and Discussion. In the Results, consider opening paragraphs by posing a biological question or hypothesis, rather than citing a phenotype. Currently, the paper is

a laundry list of phenotypes and factors (factors with similar names no less!), which is confusing to all but the most devoted C. elegans chromatin jocks. In addition, readers often have an easier time with a conclusion like gene X does a, b, c instead of loss of gene x leads to x, y z or loss of a, b, c. In the Abstract and Results, most of the description is loss of function phenotypes, which may lose people with less genetics than the authors. A summary sentence explaining the final model would be helpful. Along the same line, cartoons added to figures in the main results may help readers understand the nature of mutations, hypotheses, experimental plans etc.

1) The text in the abstract, introduction, results and discussion sections was modified and can be followed in the "track changes" document.
2) A summary sentence on the model was added in line 376.
3) Nature of mutations: a scheme for *ulp-2(tv380)* mutation has been published in Figure S2 PMID: 26412237. Schemes for all other alleles are shown in the supplemental figures and are now better referred to along the text of the revised manuscript.
4) Hypothesis and experimental plans were better explained along the revised text and schemes were added in Figure 3.

Major Points

1. Figure 1C and Fig 3C appears to show the same data for some specific genotypes. Eliminate one of these panels or use another independent dataset to represent independent trials in the two figures

We eliminated the data that appears in Figure 1C from Figure 3C. The datasets appear now in S1 data according to the panel in each figure. The summary table is in S1 data in sheet Fig.1C-3C.

2. Figure 2E. Could the authors please confirm the germ line to soma transformation using one or more endogenous markers. The days of transgenes, which can show faulty expression, is drawing to a close. Crispr alleles, smFISH, antibodies to endogenous proteins. Any of these would be more convincing, and at least Crispr and smFISH are technically easy to do.

We performed the analysis with the UNC-13::GFP reporter tagged endogenously with CRISPR/Cas9 and revealed ectopic germline expression. This new data appears in the revised manuscript in Figure S4.

3. Figure 2G. To what extent is the loss of H3K27me3 driving the phenotype of the enhanced sumoylation? Can these phenomena be separated? Similarly, the role of SET-26 binding to H3K4me3 as shown in figure 5, is this a H3K27me effect? Additional experiments would be welcome, but if not, at least talk about it in the Discussion.

Based on this comment we think that the H3K27me3 modification data is confusing in this context and do not add relevant information to this study that is focused on H3K4me3. We decided to keep the data only on the effect of *ulp-2* mutant on global H3K4me3 levels as it is the modification that is recognized by SET-26 (PMID: 29714684). This data now appears in Figure 5A.

4. "Synergy" in genetic interactions can be hard to interpret. No additivity can mean two genes function in the same genetic pathway whereas additivity can mean independent pathways or roles. This is an issue for the genetics of ulp-2 with the SET domain proteins. If SET-26 is in the same pathway, why does it synergize? Perhaps one stronger conclusion is that both factors act positively ie in the same direction, for viability and fertility. I suggest the authors rework their interpretations here. They cannot rule out a function in the soma, for example.

We agree that synergy can be hard to interpret. In this case, the synergy is observed strongly in the F1 generation, where there is still partial ULP-2 activity. In such a case, elimination of a second gene in the same pathway can be expected to enhance the phenotype. We would therefore expect that a ULP-2 target does synergize with ULP-2 (especially since SET-26 is likely not the only target so would also not result in an ULP-2 null phenotype by itself).

We also agree with the reviewer that we cannot rule out a possible function in the soma. Accordingly, we have rephrased this section in line 258: "In double mutants we observed a shorter extension of lifespan than observed in either single mutant (Figure 3D, S1 data). This suggests that while in the germline both factors act positively to maintain the germline, in the soma their mode of interaction is possibly indirect and involves additional mechanisms. "

5. Minor points

1. Is the difference between F1 animals and F2 a maternal contribution. Please test or comment on the possibility that ULP-2 is partially maternally rescued.

We performed RT-qPCR on *ulp-2(tv380)* homozygous mutant animals and WT and found that the *ulp-2* transcript levels were reduced relative to WT by ~70% and were detected in both the F1 and F2 generations. This reflects a maternal contribution in both generations. We suggest: (1) the *ulp-2* RNA is highly stable and (2) during oogenesis in the heterozygous P0 mother gonad, both maternal *ulp-2* transcript and ULP-2 protein are deposited in the oocytes and are sufficient for the development of its homozygous F1 progeny to fertile adults. The ULP-2 protein and mRNA could not be provided in the same levels by the homozygous F1 mother to its oocytes because only maternal RNA from the heterozygous P0 mother was contributed to the F1 mothers. We indicate the maternal contribution in the text in line 135.

2. Fig 2A. RNAseq fold change of 1.2 is a low threshold for gene changes, especially with such a strong increase in sumoylation. Please use a more stringent cut-off or explain why such a lenient threshold was necessary.

Strong increase in global sumoylation does not necessarily reflect a complete loss of regulation on gene transcription. The increase we observed in SUMO levels suggests that ULP-2 is a central SUMO protease in *C. elegans* and therefore may have multiple targets (hundreds) influenced in different ways and severity and together contribute to the phenotype. We chose a reduction or increase in 20% of the transcripts quantity relatively to WT as a cut off value to not exclude mild effects that would contribute to the final phenotype. This low cut off allowed us to have more confidence in the biological processes identified as impaired upon *ulp-2* loss of function.

3. Fig 2C. These images, and the arrows indicating abnormal sperm, are two small to see. Please enlarge them or include zoom in panels that makes clear the phenomenon described.

The images were enlarged and re-labeled and insets of the proximal gonad were added in the revised Figure 2C.

4. Fig2D. There are gaps in the boxplots for pachytene and diplotene. These gaps are not explained. Please address what these gaps in the data represent.

The y-axis of this graph is divided into two scales to reduce the dimension of the graph. The values are unaltered and the gaps in the box plots are the same as in the axis. The reason to present it this way is the big difference in the number of nuclei in the mitotic and transition zone relatively to diplotene and diakinesis. We cut the y-axis to allow clear visualization of the differences between the bars. We added to the figure legends of Figure 2D in line 222: "Lines on the Y axis indicate a scale change to allow observation of the quantification".

5. Fig 3C. Please comment on the suppression of set-26 mutant longevity seen in the double mutants. Are they 'ulp-2 F1 animals'. If so, please include the 'F2s' in this plot as well or address the genetic relationship in an independent manner.

The double mutant animals are the F1 *ulp-2* mutants, now labeled in the revised figure 3C. The F1 animals become sterile when both mutant alleles of *ulp-2* and *set-26* are combined as shown in Figure 3B and there is no viable F2 generation. We suggest that also in the soma ULP-2 regulates multiple factors (both negatively and positively) and that the double mutant exhibit suppression of the *set-26* mutant longevity phenotype as a result of all the changes in protein activities that occur in the soma due to aberrant desumoylation. In contrast, the sterility observed in *ulp-2* mutant animals one generation earlier when combined with a *set-26* mutant allele suggests that SET-26 activity is crucial to allow germline survival when maternal levels of ULP-2 are still expressed. The functional interaction between ULP-2 and SET-26 is further supported by our findings that increased sumoylation of SET-26 reduces its reader activity which suggests that balanced sumoylation is essential for its activity.

6. Fig S3B/C. These data are not interpretable because the control WT+ "SET-3" RNAi is missing. Please perform this control and add it to your figures to help the reader interpret the relationship between these set genes and fertility.

We repeated this experiment with the missing controls. The new results appear now in the revised Figure S5B. The RNAi experiment in this revision was made using the feeding approach and led to milder effects; some of the hermaphrodites had very few progeny but we did not detect complete sterile hermaphrodites and therefore panel S3C was removed from this revised manuscript.

7. Fig S3A and F. These graphs are unreadable. Please increase font size eg. similar to your axes in S3B/C

The fonts in the revised Figure were increased.

8. Errors and error bars in the text and figures need to be clarified. Are they standard deviation or standard error?

All errors and error bars are standard deviation (SD). This information is stated now in the "quantification and statistical analyses" section of the methods.

9. Fig legend 3. Provide a list of the 'control pairs'.

The list of control pairs in now added in S3_table and explained in the methods section.

10. Yeast 2 hybrid experiment: please address in the text the rationale for using the N terminal domain of ULP-2 rather than the full-length or C terminus.

We used the N- terminal region of ULP-2 because it is predicted to be the region that determines its subcellular localization and the specificity to the substrates while the C-terminal contains the catalytic domain. We added this explanation with references in line 230.

11. Introduction: "whereas methylation events on histone H3 on lysine residues K9, K20, and K27 are associated with gene repression [11]." This is a typo, I think you mean H3K23me, or do you mean H4K20me?

Thanks for this comment, this was a mistake and K20 was removed from the text.

12. "although we did not observe a meaningful intersection with the upregulated genes (R.F.=8)," Please define what meaningful means here (explain RF number).

Thanks for this comment, this was a typo and the R.F. value in line 327 is 0.8.

R.F. means “Representation Factor” (detailed in Figure 2B). It is used to quantify the observed intersection between two different groups relative to the expected quantity of intersection by random chance of the same two groups in the Fischer’s exact statistical test. We meant that we found no significant difference from the random chance of intersection. We have removed the word "meaningful" from this sentence and replace by "significant".

13. Among genes and proteins found in the screens, is there anything to explain the very specific meiosis phenotype?

In the group of downregulated genes we identified the following GO terms of meiosis:
meiosis I cell cycle process (GO:0061982): 60 genes
meiosis II cell cycle process (GO:0061983): 30 genes
Between the upregulated genes, there are no genes that belong to these groups.

14. Fig 5A. Indicate in the figure and legend that the protein source for the immunoblot is an IP of SET-26::GFP.

This is indicated in revised Figure 5B on the left side. We indicated it also in panel B of the Figure legend.

15. Fig 6. Move model from supplement to here.

The model was moved to Figure 6E.

16. Note that H3K27me also has a role for embryonic plasticity (Yuzyuk, Dev Cell, 2009).

We thank the reviewer for indicating this reference on germline-independent role of MES-2 that we added in the discussion in line 422.

17. Fig S2A. Please explain what the different colors mean (red vs orange) and how that differs to blue in S2B.

The colors mean to highlight the biological processes that we have focused on in the study: red was chromatin related, orange was cell cycle related, and blue was somatic neuronal related. As this labeling is confusing, we have modified to gray colors in the revised figure (Figure S3).

18. Fig S3A. Add the region of RNAi homology as a bar over the three genes (or equivalent)

The region of RNAi homology was marked above each of the genes, now appears in Figure S5A.

19. Fig S4 lacks A and B designation of the two panels. Please add.

The A and B panels were added, now Figure S6.
